# Genomic background and genetic relationships between boar taint and fertility traits in German Landrace and Large White

**DOI:** 10.1186/s12863-020-00865-z

**Published:** 2020-06-08

**Authors:** Ines Brinke, Christine Große-Brinkhaus, Katharina Roth, Maren J. Pröll-Cornelissen, Hubert Henne, Karl Schellander, Ernst Tholen

**Affiliations:** 1 Institute of Animal Science, University of Bonn, 53115 Bonn, Germany; 2 Association for Bioeconomy Research (FBF e.V.), Adenauerallee 174, 53113 Bonn, Germany; 3 BHZP GmbH, An der Wassermühle 8, 21368 Dahlenburg-Ellringen, Germany

**Keywords:** Boar taint, Reproduction, Pigs, Genome wide association analysis, Androstenone, Skatole

## Abstract

**Background:**

Due to ethical reasons, surgical castration of young male piglets in their first week of life without anesthesia will be banned in Germany from 2021. Breeding against boar taint is already implemented in sire breeds of breeding organizations but in recent years a low demand made this trait economically less important.

The objective of this study was to estimate heritabilities and genetic relationships between boar taint compounds androstenone and skatole and maternal/paternal reproduction traits in 4′924 Landrace (LR) and 4′299 Large White (LW) animals from nucleus populations. Additionally, genome wide association analysis (GWAS) was performed per trait and breed to detect SNP marker with possible pleiotropic effects that are associated with boar taint and fertility.

**Results:**

Estimated heritabilities (h^2^) were 0.48 (±0.08) for LR (0.39 ± 0.07 for LW) for androstenone and 0.52 (±0.08) for LR (0.32 ± 0.07 for LW) for skatole. Heritabilities for reproduction did not differ between breeds except age at first insemination (LR: h^2^ = 0.27 (±0.05), LW: h^2^ = 0.34 (±0.05)). Estimates of genetic correlation (r_g_) between boar taint and fertility were different in LR and LW breeds. In LR an unfavorable r_g_ of 0.31 (±0.15) was observed between androstenone and number of piglets born alive, whereas this r_g_ in LW (− 0.15 (±0.16)) had an opposite sign. A similar breed-specific difference is observed between skatole and sperm count. Within LR, the r_g_ of 0.08 (±0.13) indicates no relationship between the traits, whereas the r_g_ of − 0.37 (±0.14) in LW points to an unfavorable relationship. In LR GWAS identified QTL regions on SSC5 (21.1–22.3 Mb) for androstenone and on SSC6 (5.5–7.5 Mb) and SSC14 (141.1–141.6 Mb) for skatole. For LW, one marker was found on SSC17 at 48.1 Mb for androstenone and one QTL on SSC14 between 140.5 Mb and 141.6 Mb for skatole.

**Conclusion:**

Knowledge about such genetic correlations could help to balance conventional breeding programs with boar taint in maternal breeds. QTL regions with unfavorable pleiotropic effects on boar taint and fertility could have deleterious consequences in genomic selection programs. Constraining the weighting of these QTL in the genomic selection formulae may be a useful strategy to avoid physiological imbalances.

## Background

Boar taint is described as an unpleasant smell of the meat from entire male pigs [1], which occurs as soon as the young pigs reach puberty. There are two main compounds which are responsible for boar taint. The first one is androstenone (5α-androst-16-en-3-one) [2], a steroid hormone which is built in the Leydig cells of the testis. The second one is skatole (3-methyindole) which results from the degradation of the amino acid tryptophan in the colon [3]. Both compounds can be affected by genetics and environmental factors whereas skatole is more sensitive to housing conditions and nutritional management [4, 5]. Currently, surgical castration without anesthesia is performed on young male piglets in their first week of life to prevent that odor, which represents a strong contrast to the increasing role of animal welfare in consumer acceptance. Due to a modification of the German animal protection law in 2013, castration without anesthesia should have been banned in Germany from 2019 but disagreement about alternatives lead to an extension of the deadline for the ban for two more years until 2021 [6].

When it comes to the integrity of the animal, fattening of entire boars is a suitable option to replace surgical castration. Furthermore, raising of entire males can be more sustainable regarding feed conversion, carcass composition [7] and carbon footprint [8]. To establish this method as a long-term alternative, it is necessary to reduce the percentage of odorous boars at slaughterhouse. This can be achieved by breeding against boar taint, as previous reported h^2^ showed a genetic potential of both compounds [9]. As has been suggested by some breeding organizations, boar taint is included into the breeding goal of selected sire breeds [10–12]. Information about an implementation of boar taint into breeding objectives of maternal nucleus populations cannot be found which indicates that there have been no activities in selection against boar taint.

Due to high genetic correlations between the boar taint compound androstenone and steroid hormones like testosterone, estrone sulfate and 17β-estradiol [13–15] antagonistic relationships between boar taint and fertility traits have to be expected. This is supported by common physiological pathways of androstenone and steroid hormone synthesis [16]. As reproduction represents an economically important trait, especially in maternal nucleus populations, breeding against boar taint could lead to a deterioration of traits from recent breeding goals in female reproduction traits like the number of piglets born alive or age at first insemination as well as in male reproduction traits [17]. Negative relationships between boar taint and paternal fertility traits like the length of bulbourethral gland as an indicator for sexual maturation in boars has been reported by Tajet et al. [18]. Additionally, high correlations between androstenone and physiologically linked sex hormones like testosterone were found by Grindflek et al. [14] which indicate possible antagonisms to paternal fertility. However, in contrast to these results Strathe et al. [19] have estimated favorable genetic correlations between boar taint compounds and different semen traits. In a similar way impact breeding of against boar taint compounds on maternal fertility is still under discussion due to controversial results [1, 17, 20]. As common synthesis and high correlations affirm an interrelated control by genomic regions [15], it is important to identify genes or regions with a stimulating influence on androstenone/skatole degradation without adverse effects on both, male and female fertility [14].

Therefore, the aim of this study was to investigate the relationship between boar taint compounds and reproduction traits by estimating genetic correlations and heritabilities in Landrace (LR) and Large White (LW) populations. Additionally, genome wide association analysis (GWAS) was performed per trait and breed to detect SNP marker with possible pleiotropic effects that are associated with boar taint and fertility.

## Methods

### Phenotypes

All phenotypes related to boar taint, maternal and paternal reproduction traits were recorded within a LR and LW nucleus population of a commercial breeding organization, respectively. Pedigree information was available for all animals up to 18 generations in both breeds. The LR pedigree contained 3′331 males and 3′967 females with an average inbreeding coefficient of 0.019. The LW pedigree contained 2′410 males and 3′122 females with an average inbreeding coefficient of 0.021.

### Boar taint

Purebred LR- and LW-boars were raised under the same conditions in a central testing station. A total of 1′410 LR and 1′396 LW boars was slaughtered at a constant age of 160 days in the routine process of a commercial EU-certificated abattoir. Animals were anesthetized using a 92% CO_2_ atmosphere and bled by cutting the main arteries closer to the heart. Tissue samples were collected at birth for DNA extraction and genotyping. Adipose tissue samples were collected post-slaughter from the neck area at slaughterhouse and stored at − 20 °C until analysis. Androstenone (AND) and skatole (SKA) concentration in adipose tissue was analyzed in all samples by using a standardized stable isotope dilution analysis-headspace solid-phase microextraction-gas chromatography/mass spectrometry (SIDA-HSPM-GC/MS) [21]. Because of the skewness of AND and SKA, concentrations were log-transformed into log_AND and log_SKA for all statistical analyses. Estimated heritabilities and GWAS regarding boar taint are based on these log-transformed values.

### Maternal reproduction

Maternal reproduction traits included information about number of piglets born alive (NBA), number of piglets born dead (NBD) and age at first insemination (AFI) and was routinely collected from 2′049 (LR) and 2′096 (LW) sows in 4′519 (LR) and 5′205 (LW) litters. Information about AFI was provided for 1′529 LR and 1′866 LW sows.

### Paternal reproduction

Paternal reproduction information comprised the traits sperm volume (SV), sperm density measured by photometer (SP) and sperm count in billions (SC) and was collected from 1′465 (LR) and 807 (LW) boars with 41′104 (LR) and 21′935 (LW) manual observations at insemination stations.

Animal care within all herds followed the general guidelines outlined in the European animal welfare regulations.

### Variance component estimation

Variance components were estimated with a multivariate approach using ASReml® [22]. Analyzed traits log_AND, log_SKA, NBA, NBD, AFI, SV, SC and SP were evaluated in a full multiple eight trait model in combination with the pedigree information. Residual covariance between traits that cannot be measured in the same individual like paternal and maternal fertility were fixed to 0. Breeds were analyzed separately.

Variance components were estimated by using the following polygenetic model for the boar taint compounds log_AND and log_SKA:

1 $$ y= X\beta +{Z}_1u+{Z}_2w+e $$

where y contains the observed traits. The generalized linear mixed model (Eq. 1) was fitted to log_AND and log_SKA and consisted of year-season of slaughter (37 levels in LR and LW) as fixed environmental effect denoted by the vector *β* and animal, pen and error as random effects, represented by the vectors *u*, *w* and *e*, respectively. Weight and age at slaughter were used as covariates in this model. e is the vector of random residual effects. **X**, **Z**_1_ and **Z**_2_ were the corresponding incidence matrices for the fixed effects in *β* and the random effects *u* and *w*, respectively.

Reproduction traits with repeated measurements are estimated by using a polygenetic model including the repeated measurements (*pe*) as a random effect:

2 $$ y= X\beta +{Z}_1u+{Z}_3 pe+e $$

Equation 2 for the maternal reproduction traits consisted of herd-year-season (130 levels in LR, 44 levels in LW) of litter as a fixed environmental effect represented by vector *β* and animal (*u*) and error (*e*) as random effects. Additionally, for the traits NBA and NBD litter number was included as a fixed effect in the model. Repeated measurements per sow were considered as a random effect for NBA and NBD in vector pe.

Equation 2 for the paternal reproduction traits consisted of herd-year-season of sperm sample date (58 levels in LR and LW) and station (three levels in LR and LW) as fixed environmental effects and animal as a random effect. Age of the boar at sample date was used as covariate in the model. Repeated measurements per boar were included as an additional random effect (*pe*).

For Eq. 2, **X**, **Z**_1_ and **Z**_3_ were handled as the incidence matrices for the fixed effects in* β* and the random effects *u* and *pe*, respectively.

### Genotype data

A total of 2′729 (LR) and 2′908 (LW) pigs were also genotyped by the Illumina PorcineSNP60 BeadChip (Illumina, San Diego, CA, USA). Details about the number of genotyped animals per breed, trait and sex are reported in Table 1. This data was used to perform a GWAS for boar taint compounds and reproduction traits, separated by trait and line.

**Table 1:** Number of genotyped animals for GWAS per trait and breed

Complex	Trait	Number of animals	Number of observations	Markers	Breed
Boar taint	log_AND, log_SKA	1′293	1′293	38′411	LR
		1′317	1′317	39′302	LW
Female reproduction	NBA, NBD	1′083	2′932	38′532	LR
		1′282	3′476	39′442	LW
Female reproduction	AFI	961	961	38′504	LR
		1′267	1′267	39′450	LW
Male reproduction	SV, SC, SP	353	11′675	37′991	LR
		309	6′913	39′089	LW

*log_AND* log-transformed androstenone, *log_SKA* log-transformed skatole, *NBA* number of piglets born alive, *NBD* number of piglets born dead, *AFI* age at first insemination, *SV* sperm volume, *SC* sperm count in billions, *SP* sperm density measured by photometer.

SNPs and individuals with a call-rate of less than 0.95 and SNPs with a minor allele frequency (MAF) less than 0.05 were excluded from further analysis. The quality control was conducted with PLINK [23]. For further analysis, 2′729 LR and 2′908 LW pigs with a marker amount between 37′991 and 39′450 SNPs, depending on the trait were available. Information about the number of animals and markers per trait that was available for GWAS after quality control are shown in Table 1.

### GWAS

GWAS was performed with the R-package GenABEL [24]. Within the GWAS log-transformed concentrations were regarded as a phenotype for AND and SKA. Because GenABEL [24] allows only one record per animal, we have calculated an adjusted mean per sow / boar for the reproduction traits with repeated measurements (NBA, NBD, SV, SC, SP). This calculation was performed by using Eq. 2, excluding the additive genetic effect. The resulting pe-effects of the sows / boars were interpreted as such an adjusted mean per sow / boar and were used as a new phenotype for GWAS analysis. For AFI, the raw phenotype was used.

Due to the recording and selection scheme, the sample size and structure for the trait complexes boar taint and reproduction differ. As a result, different levels of population stratification within these datasets can be observed. For AND and SKA all analyzed animals were randomly selected from the population. In both resulting LR/LW datasets, population stratification was unexplainable moderate to high as indicated by λ-values > 2.5. In order to correct for this detrimental effect the GRAMMAR approach [25] was applied. After correction, the λ-values were in an acceptable range between 1.0 and 1.05. As a first step of the GRAMMAR approach, phenotypic data was corrected as described in Eq. 1 under consideration of genomic kinship matrix. Genomic kinship was estimated by implemented functions in the GenABEL package [24]. Resulting residuals from this model can be used as new phenotypes for the following association studies.

The reproduction traits were displayed by animals from the nucleus population, which represents a preselected sample set. Within these data sets the λ-values were low to moderate (< 1.5). In this situation, the genomic control (GC) approach by Devlin and Roeder [26] was regarded as sufficient to correct for the population stratification. The following formula was applied:

$$ {T}_{corrected}={\frac{T}{\lambda}}^2, $$

whereas T^2^ is the empirical test statistic for each locus by a fast score test or t-test and λ is the value of population stratification. Resulting *p*-values were transformed by Bonferroni correction to avoid error accumulation by multiple testing. Markers with an adjusted *p-*value < 0.05 were handled as genome wide/chromosome wide significant. Additionally, the variance explained by the single SNP was calculated according to the transformation of the student’s *t*-distribution into a *z*-distribution [27] using following formula:

$$ Var\left[\%\right]=\frac{\chi_{1 df}^2}{N-2+{\chi}_{1 df}^2}, $$

whereas ${\chi}_{1 df}^2$ is the test statistic of each SNP from GWAS and N the number of animals. Locations of SNPs for the analysis are in accordance with the recent pig genome sequence SusScrofa 11.1, variants are identified according to Ensembl release 95 [28].

## Results

The number of animals, overall means and standard deviations of raw phenotypes and log-transformed data are shown in Table 2 for LR and LW, respectively. Animals were slaughtered at a mean age of 163.6 days (LR) and 165.2 days (LW). The average slaughter weight was 94.5 kg for LR and 88.9 kg for LW.

**Table 2:** Descriptive statistics of the analyzed traits

Trait	LR			LW		
	N	Mean	SD	N	Mean	SD
AND (ng/g in fat)	1′410	1883.72	1269.90	1′396	1284.90	1021.87
log_AND	1′410	7.32	0.69	1′396	6.90	0.73
SKA (ng/g in fat)	1′410	183.89	156.80	1′396	82.10	89.96
log_SKA	1′410	4.88	0.82	1′396	4.10	0.72
NBA	2′049	14.75	3.24	2′096	14.52	3.74
NBD	2′049	1.48	1.68	2′096	0.89	1.44
AFI (days)	1′529	254.71	13.22	1′866	274.75	53.39
SV (ml)	1′465	209.68	77.69.26	807	237.09	76.94
SC (count in billions)	1′465	62.91	22.67	807	62.66	22.60
SP (OD)	1′465	394.94	143.34	807	340.68	113.54

*AND* androstenone, *log_AND* log-transformed androstenone, *SKA* skatole, *log_SKA* log-transformed skatole, *NBA* number of piglets born alive per litter, *NBD* number of piglets born dead per litter, *AFI* age at first insemination, *SV* sperm volume, *SC* sperm count in billions, *SP* density of sperm measured by photometer (SP) in optical density (OD).

### Variance component estimation

In general, estimated heritabilities and genetic correlations in this study are based on the log-transformed value of AND and SKA and were not transformed in its original scale. Variance component estimation (Table 3) showed moderate to high h^2^ of 0.50 for log_AND in LR (h^2^ = 0.39 in LW) and of 0.52 for log_SKA in LR (h^2^ = 0.32 in LW). Phenotypic correlations (r_p_) between log_AND and log_SKA were similar (r_p_ = 0.30) in both breeds whereas genetic correlations (r_g_) were slightly different (r_g_ = 0.29 in LR and r_g_ = 0.41 in LW).

**Table 3:** h^2^, r_g_ and r_p_ for boar taint compounds and maternal reproduction traits (LR and LW)

	log_AND		log_SKA		NBA		NBD		AFI		Breed
log_AND	**0.50**	**(0.08)**	0.29	(0.12)	0.31	(0.15)	0.00	(0.16)	−0.10	(0.15)	LR
	**0.39**	**(0.07)**	0.41	(0.14)	−0.15	(0.16)	0.15	(0.19)	0.01	(0.14)	LW
log_SKA	0.32		**0.52**	**(0.08)**	0.18	(0.15)	0.04	(0.16)	0.36	(0.14)	LR
	0.25		**0.32**	**(0.07)**	−0.25	(0.16)	0.06	(0.21)	− 0.34	(0.14)	LW
NBA	0.61		0.47		**0.12**	**(0.03)**	0.34	(0.14)	0.16	(0.13)	LR
	0.19		0.15		**0.14**	**(0.03)**	0.36	(0.13)	0.06	(0.10)	LW
NBD	0.14		0.12		0.14		**0.09**	**(0.02)**	0.14	(0.14)	LR
	0.12		0.09		0.00		**0.07**	**(0.02)**	0.38	(0.13)	LW
AFI	−0.04		0.13		0.01		0.02		**0.27**	**(0.05)**	LR
	0.00		−0.11		0.00		−0.01		**0.34**	**(0.05)**	LW

h^2^ (± standard error) on the diagonal, *r*_*p*_ phenotypic correlation under the diagonal, *r*_*g*_ genetic correlation above the diagonal, *log_AND* log-transformed androstenone, *log_SKA* log-transformed skatole, *NBA* number of piglets born alive per litter, *NBD* number of piglets born dead per litter, *AFI* age at first insemination.

Heritabilities for NBA and NBD were in a range of 0.07 to 0.14 in both breeds (Table 3). For AFI, h^2^ was 0.27 for LR and 0.34 for LW. Genetic correlations between NBA and NBD and NBA and AFI did slightly differ between the breeds. In contrast to that, the r_g_ of NBD and AFI was nearly three times higher in LW (r_g_ = 0.38) than in LR (r_g_ = 0.14) with high standard errors in both breeds. The permanent environmental effect (pe^2^) of the sow was low with 0.10 for NBA in LR (pe^2^ = 0.04 in LW) and 0.05 for NBD in LR (pe^2^ = 0.04 in LW).

Heritabilities for sperm quality traits were mainly high in a range from 0.39 to 0.48 in both breeds (Table 4). High positive r_g_ between SV and SC of 0.51 in LR and 0.54 in LW showed that an increase in sperm volume would result in an increase of sperm count. The sperm density was genetically highly positive correlated with the sperm count in both breeds. An increase in sperm count would hence result in a higher density of the ejaculate.

**Table 4:** h^2^, r_g_ and r_p_ for boar taint compounds and paternal reproduction traits (LR and LW)

	log_AND		log_SKA		SV		SC		SP		Breed
log_AND	**0.50**	**(0.08)**	0.29	(0.12)	−0.18	(0.13)	−0.17	(0.14)	0.03	(0.03)	LR
	**0.39**	**(0.07)**	0.41	(0.14)	−0.25	(0.14)	−0.19	(0.15)	0.04	(0.15)	LW
log_SKA	0.32		**0.52**	**(0.08)**	0.04	(0.13)	0.08	(0.13)	0.06	(0.13)	LR
	0.25		**0.32**	**(0.07)**	0.08	(0.14)	0.37	(0.14)	0.32	(0.14)	LW
SV	0.16		0.21		**0.46**	**(0.01)**	0.51	(0.02)	−0.55	(0.02)	LR
	0.22		0.32		**0.44**	**(0.02)**	0.54	(0.03)	−0.44	(0.04)	LW
SC	−0.05		0.05		0.57		**0.43**	**(0.01)**	0.43	(0.03)	LR
	0.06		0.25		0.59		**0.39**	**(0.02)**	0.50	(0.04)	LW
SP	0.63		0.51		−0.40		0.60		**0.45**	**(0.01)**	LR
	0.83		0.84		−0.31		0.69		**0.48**	**(0.02)**	LW

h^2^ (± standard error) on the diagonal, *r*_*p*_ phenotypic correlation under the diagonal, *r*_*g*_ genetic correlation above the diagonal, *log_AND* log-transformed androstenone, *log_SKA* log-transformed skatole, *SV* sperm volume, *SC* sperm count in billions, *SP* sperm density (measured by photometer).

As shown in Table 3 genetic correlation between log_AND and NBA is moderate to low in LR (r_g_ = 0.31) and LW (r_g_ = − 0.15) but different in the sign. As a consequence, breeding against AND would result in a lower NBA in LR and a higher NBA in LW. The r_g_ between log_SKA and AFI shows another distinct difference between the breeds. While breeding against SKA seems to extend the AFI in LW (r_g_ = − 0.34), this is the opposite in the LR breed where the correlation is moderately positive (r_g_ = 0.36).

Favorable genetic relationship was observed between log_AND and SV within both breeds (LW: r_g_ = − 0.18, LW r_g_ = − 0.25). In contrast, regarding the r_g_ between log_SKA and SC breeding against SKA might have unfavorable consequences for paternal fertility. However, the undesired outcomes for SC are more relevant within the LW (r_g_ = 0.37) than within the LR breed (r_g_ = 0.08). Similar results are observed in the r_g_ between log_SKA and SP, where the r_g_ of 0.32 in LW points to an unfavorable consequence for paternal fertility, whereas the r_g_ between these traits in LR is near 0 (r_g_ = 0.06).

Besides the genetic correlation between boar taint and fertility traits some other relationships between paternal and maternal fertility traits are worthwhile to mention (Table 5). While r_g_ between SC and AFI is close to zero in LR (r_g_ = 0.09), these traits are moderately negative correlated in LW (r_g_ = − 0.34). Another noticeable breed difference is observed regarding the genetic correlation between SP and AFI. These estimates suggest that (indirect) breeding against sperm count or sperm density result in a later AFI in LW, whereas it shortens the AFI in LR. Genetic correlation between SV and NBD also indicate breed differences. Indirect breeding against SV could result in a lower NBD (r_g_ = 0.21) in LR, whereas no consequences in the LW can be expected as indicated by the estimated genetic correlation coefficient (r_g_ = − 0.07).

**Table 5:** h^2^, r_g_ and r_p_ for paternal and maternal reproduction traits (LR and LW)

	SV		SC		SP		NBA		NBD		AFI		Breed
SV	**0.46**	**(0.01)**	0.51	(0.02)	−0.55	(0.03)	− 0.14	(0.12)	0.21	(0.14)	−0.11	(0.12)	LR
	**0.44**	**(0.02)**	0.54	(0.03)	−0.44	(0.04)	−0.26	(0.11)	−0.07	(0.15)	−0.05	(0.12)	LW
SC	0.57		**0.43**	**(0.01)**	0.43	(0.03)	0.27	(0.13)	0.26	(0.15)	0.09	(0.12)	LR
	0.59		**0.39**	**(0.02)**	0.50	(0.04)	0.04	(0.12)	0.11	(0.16)	−0.34	(0.12)	LW
SP	− 0.40		0.60		**0.45**	**(0.01)**	0.40	(0.12)	0.06	(0.14)	0.26	(0.12)	LR
	−0.31		0.69		**0.48**	**(0.02)**	0.53	(0.10)	0.27	(0.15)	−0.25	(0.12)	LW
NBA	0.05		0.20		0.23		**0.12**	**(0.03)**	0.34	(0.14)	0.16	(0.13)	LR
	0.01		0.09		0.28		**0.14**	**(0.03)**	0.36	(0.13)	0.06	(0.10)	LW
NBD	0.15		0.09		0.25		0.14		**0.09**	**(0.02)**	0.14	(0.14)	LR
	0.13		0.10		0.38		0.00		**0.07**	**(0.02)**	0.38	(0.13)	LW
AFI	−0.03		0.03		0.13		0.01		0.02		**0.27**	**(0.05)**	LR
	−0.01		−0.12		− 0.09		0.00		−0.01		**0.34**	**(0.05)**	LW

h^2^ (± standard error) on the diagonal, *r*_*p*_ phenotypic correlation under the diagonal, *r*_*g*_ genetic correlation above the diagonal, *SV* sperm volume, *SC* sperm count in billions, *SP* sperm density (measured by photometer), *NBA* number of piglets born alive, *NBD* number of piglets born dead, *AFI* age at first insemination.

### GWAS

A summary of significant associated markers per trait along with their position are presented in Additional File 1 for LR (see Supplementary Material, Additional File 1) and Additional File 2 for LW (see Supplementary Material, Additional File 2). In total, 28 markers in LR and 18 markers in LW were found to be significantly associated with log_AND, log_SKA, AFI and NBD. For all other reproduction traits, no significant markers were identified.

### Androstenone

**Fig. 1:**
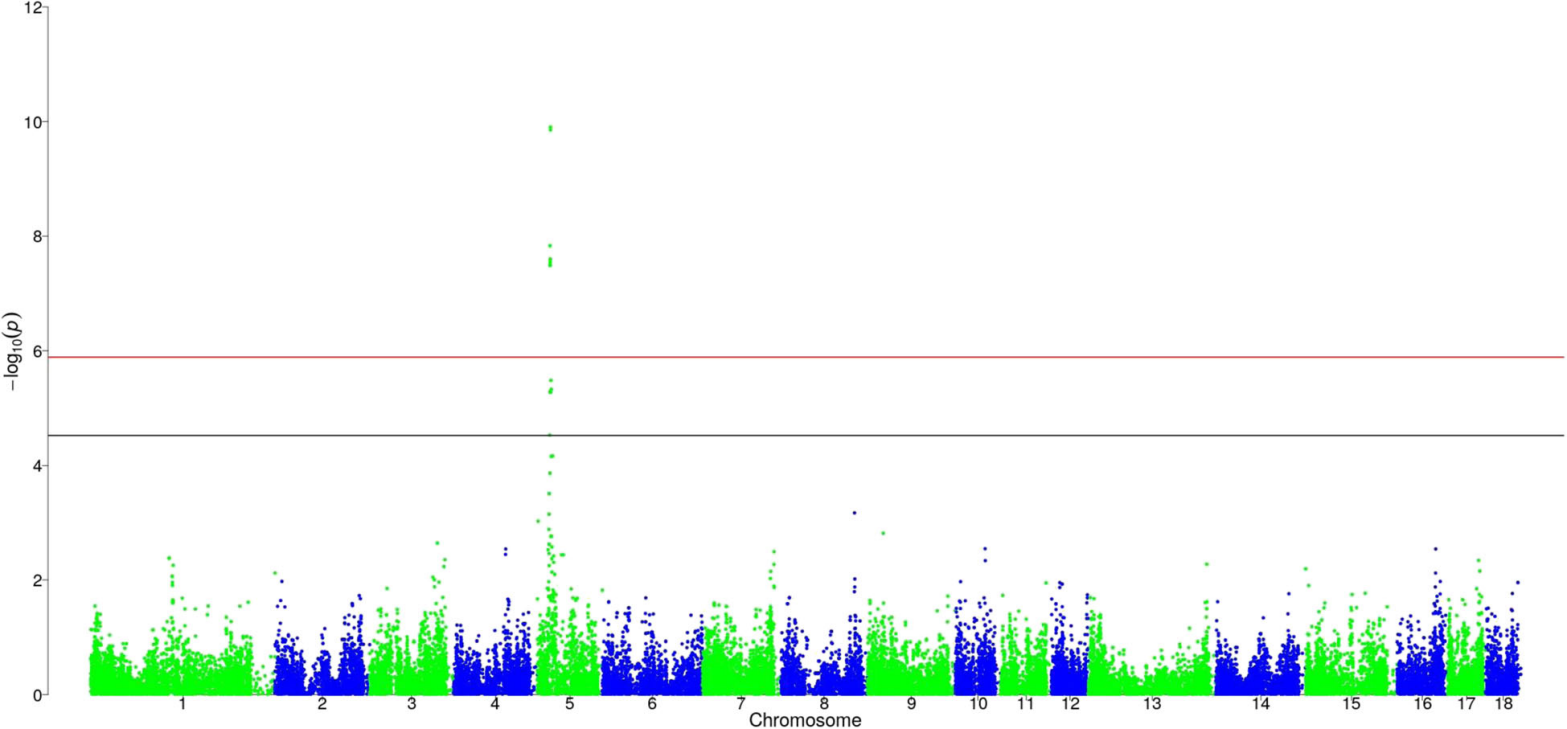
Distribution of SNPs for log-transformed androstenone in Landrace. Black line corresponds to the threshold of chromosome wide significance, red line corresponds to the threshold of genome wide significance

Androstenone in LR was found to be significantly associated with nine genome wide significant markers (Fig. 1). Additionally, 5 markers were also chromosome wide significant. Two of these markers were not mapped until now. The most important region was identified on *Sus scrofa Chromosome* (SSC) 5 and is ranging from 20.9 Mb to 22.9 Mb. It contains 12 significant SNPs of which five were intron variants, one was an upstream gene variants, one was a downstream gene variant and one was a splice region variant as well as one synonymous, one 3′ prime untranslated region (3’PUTR) variant and two intergenic variants. Phenotypic variance explained by a significant SNP in this region varied between 1.3 and 3.1%.

In LW one marker was found to be chromosome wide significant associated for log_AND at 48.1 Mb on SSC 17. This marker is a 3′ prime untranslated region (3’PUTR) variant, explaining 1.3% of the phenotypic variance.

### Skatole

GWAS for log_SKA revealed two chromosome wide associations with markers on SSC 14 in LR (17 markers in LW). Both markers in LR and four markers in LW were also genome wide significant.

**Fig. 2:**
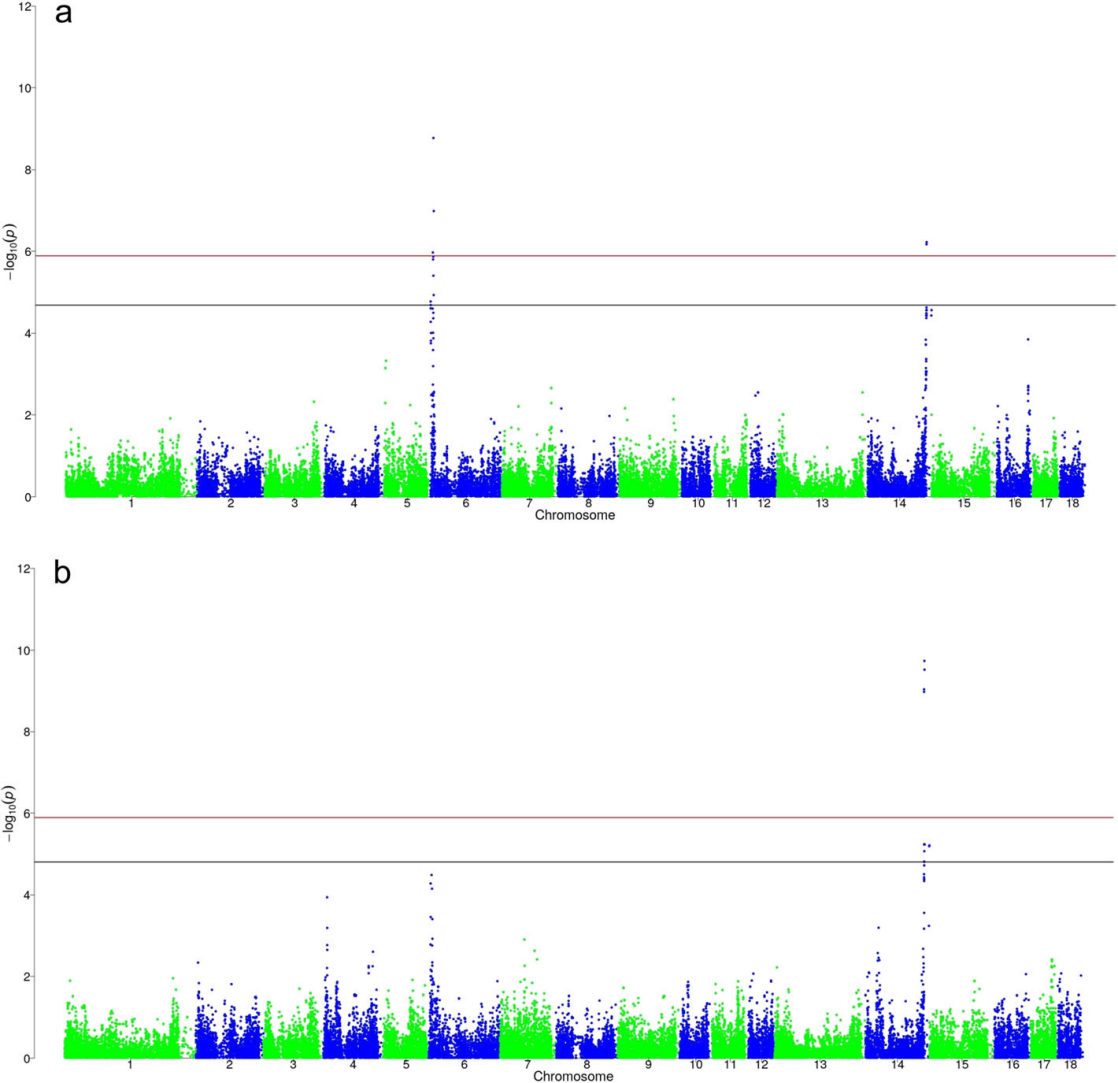
**a** Distribution of SNPs for log-transformed skatole in Landrace. Black line corresponds to the threshold of chromosome wide significance, red line corresponds to the threshold of genome wide significance. **b** Distribution of SNPs for log-transformed skatole in Large White. Black line corresponds to the threshold of chromosome wide significance, red line corresponds to the threshold of genome wide significance

All significant markers for both breeds on SSC 14 are located in a region from 140.5 Mb to 141.6 Mb (Fig. 2a, b), except for two markers in LW that were located around 153 Mb. An upstream gene variant of SNP *SIRI0000194* on SSC 14 was found to be genome wide significant for both breeds as well as an intergenic variant (*ASGA0068311)*. The variance explained by a significant SNP varied between 1.5 and 2.7%.

Additionally, nine markers were found to be chromosome wide significant associated with log_SKA in LR on SSC 6 (three of them were also genome wide significant) (Fig. 2a). These QTL were located in two delimitable regions. The first region is ranging from 0.3 Mb to 0.4 Mb containing 2 markers and the second region is ranging from 5.5 Mb to 7.5 Mb, which includes 7 markers. Explained variance by SNP in these regions was ranging from 1.3 to 2.7%.

### Maternal reproduction traits

In maternal reproduction traits, significant associations were only found for NBD and AFI in LR.

For NBD one marker was identified as chromosome wide significant on SSC 1. It is an intron variant around 92.1 Mb which explains 2.9% of phenotypic variance.

GWAS for AFI revealed two chromosome wide significant markers, one on SSC 1 and one on SSC 2. The marker on SSC 1 is at 0.4 Mb and thus, is not overlapping with the detected one for NBD. The variance explained by this significant SNP was 4.1%. The significant marker on SSC 2 is located at 11.7 Mb and its variance explained by this SNP was 2.8%.

## Discussion

The importance of animal welfare in pig production systems has increased which has led to a ban of surgical castration from 2021 in Germany. To achieve this ban, it is necessary to face alternatives like fattening of entire male pigs.

This alternative is only feasible if the amount of tainted carcasses of entire boars will be reduced close to zero. Hence, breeding against boar taint is an important and sustainable tool to reach this goal. However, unfavorable relationships between boar taint and fertility can be expected due to common endocrinological synthesis [29]. This study aims to reveal these relationships as well as identify genes or QTLs with possible pleiotropic effects on boar taint and fertility.

The descriptive data showed that the concentrations of AND and SKA in fat were on average much greater in LR (2′062 ng/g for AND, 188.5 ng/g for SKA) compared with LW (1′4*22* ng/g for AND, 77.5 ng/g for SKA).

These findings contrasts with results of Xue et al. [30] who reported higher AND concentrations in LW than in LR. Newer studies describe LR as a breed with a high AND potential [3], which can be due to the breeding history of both breeds in the past 20 years. Due to e.g. individual sensitivity or product type perception thresholds of the safe box, which indicates an acceptable low risk of boar taint can vary between < 1′500 to < 3′000 ng/g for AND and < 150 to < 250 ng/g for SKA [31]. Applying the lowest thresholds of 1′500 ng/g AND and 150 ng/g SKA, 66.2% of all LR and 33.8% of all LW boars would be classified as conspicuous. By taking into account that SKA could have a bigger impact on the perception of boar taint than AND [32], limiting the thresholds of only SKA to 150 ng/g and disregarding AND limits would result in a proportion of rejected carcasses of 41.1% in LR and 10.9% in LW.

### Genetic background for boar taint compounds

The heritabilities in the present study for log_AND (0.50 in LR; 0.39 in LW) and log_SKA (0.52 in LR; 0.32 in LW) are in accordance to reviewed ranges in the literature of 0.25 to 0.88 for AND and 0.19 to 0.54 for SKA [9, 13, 33, 34]. This wide range is caused by genetically determined differences between breeds due to growth rate, backfat thickness and sexual maturation. Further development of technique and methods of the quantification of AND and SKA could play an additional role in the estimation of h^2^.

The genetic correlation between log_AND and log_SKA was r_g_ = 0.29 in LR and around r_g_ = 0.41 in LW. The findings for LR are close to reported values of 0.35 and 0.36 [18, 34]. The genetic correlation between log_AND and log_SKA is already physiologically explained by Doran et al. [35] who described that the induction of the gene *Cytochrome P450 2E1* (CYP2E1), which is involved in the skatole metabolism can be blocked by high concentrations of AND in pig hepatocytes. As a consequence, an increasing AND concentration leads to an increasing SKA concentration, because SKA cannot be degraded by the liver anymore and accumulates in fatty tissue like backfat.

As the heritabilities of log_AND and log_SKA showed a high breeding potential for breeding against these boar taint compounds, possible negative relationships to reproduction traits have to be considered due to similar synthesis pathways [16]. To ascertain the extent of these possible unfavorable consequences, r_g_ were determined between maternal reproduction traits and boar taint compounds.

### Boar taint and maternal fertility

Low heritabilities for NBA and NBD in LR are consistent with what has been reported in the literature for LR and LW [36, 37]. Furthermore, in LR h^2^ for AFI (h^2^ = 0.27) is in accordance with the reported h^2^ of Mathur et al. [17] (AFI = 0.27). The high h^2^ of AFI in LW in this study (0.34) is more comparable with the h^2^ of Piétrain breed (h^2^ AFI = 0.34), which was also reported by Mathur et al. [17]. Some of the genetic correlations between boar taint compounds and fertility were favorable or close to zero in both breeds, like the r_g_ between log_AND and NBD or between log_AND and SP. However, some genetic correlations between boar taint compounds and fertility showed a non-consistent picture but indicated that there could be unfavorable relationships. For example, the r_g_ between log_AND and NBD in LW is unfavorable (r_g_ = 0.15) whereas in LR it is close to zero which is comparable to the correlation of 0.04 as reported by Mathur et al. [17]. Similar unfavorable genetic relationships are observed between log_AND and SC in both breeds or log_SKA and SC in LW which is in contrast to the results of Strathe et al. [19] who observed favorable relationships between boar taint compounds and semen traits.

The negative genetic correlation of − 0.34 between AFI and log_SKA in LW represent the well-known unfavorable relationship between the onset of puberty and boar taint risk [38–40] however the high standard error (SE) has to be considered in the interpretation of this result. Previous reported unfavorable relationships between log_AND and AFI [38, 40] were not confirmed. Genetic correlation between log_AND and NBD in LR is zero and slightly comparable to the correlation of 0.04 between log_AND and number of stillborn as reported by Mathur et al. [17].

### Boar taint and paternal fertility

The shared synthesis pathway of AND and sex steroid hormones like testosterone may also have consequences for paternal fertility traits [14]. Thus, testosterone as a precursor of AND is a sex hormone which is necessary for spermatogenesis in boars [41] due to its regulatory function on the *gonadotropin-releasing hormone* (GnRH) pulse frequency [42]. In the HPA axis the GnRH pulse frequency influences the release of the *luteinizing hormone* (LH) which is required for the development of paternal and maternal maturity [42]. By analyzing sperm quality parameters it has to be taken into account, that these traits are influenced to a large extent by environmental effects as age of the boar or frequency of sperm collecting [43]. Moreover, different techniques were used in the artificial insemination stations (AI-stations) to measure sperm quality parameters. As a consequence, results of the different AI-stations might have an impact on the expression of these traits. In our study estimated h^2^ for paternal reproduction traits were mainly high in a range of 0.39 to 0.48. These h^2^ are higher than the results of Wolf [44] and Strathe et al. [34] who estimated values between 0.08 and 0.20 within the purebred Czech LR and LW pig populations [44] and between 0.17 and 0.31 in Danish LR boars [34]. High h^2^ for paternal reproduction traits are observed in a Piétrain crossbred study by Frieden et al. [40]. Genetic parameters between SV and SP estimated in our study indicate a distinct antagonistic genetic relationship, which is in accordance with observations in the Czech purebred pendants in the study of Wolf [44].

In the current study, r_g_ between log_AND and sperm quality parameters do not seem to be unfavorable related in both breeds, as all correlations are moderate favorable or close to zero. That means that breeding against log_AND would not result in lower SV, lower SP or lower SC. Within the LR breed the low r_g_ between log_SKA and sperm quality parameters leads to the same conclusion as Strathe et al. [34] that breeding against SKA would not impair paternal fertility traits. The opposite can be observed regarding SKA and sperm quality parameters within the LW breed. Here, the genetic relationships between log_SKA and paternal reproduction traits are moderate to high unfavorable, which means that breeding against SKA could lower the genetic potential of SV, SC and SP. However, the high SEs of all genetic correlations between boar taint compounds and paternal fertility limit the significance of our study. In addition, it should be taken into account that our dataset does not include AI-boars with extreme negative sperm quality parameters as these boars were preselected by the AI-station.

### Maternal and paternal fertility

Estimation of genetic parameters between paternal and maternal reproduction traits like SV and NBA showed a r_g_ of − 0.14 in LR and a r_g_ of − 0.24 in LW. These findings are in contrast to previously reported correlations in an earlier study in Czech LR and LW [44] which showed an r_g_ of − 0.01 between SV and NBA in LR and an r_g_ of 0.21 for LW.

### GWAS

Quantitative analyses showed the genetic background of the analyzed trait. Additionally, GWAS was performed to reveal possible candidate genes or genes with possible pleiotropic effects on boar taint compounds and fertility. In the present study, univariate GWAS per trait and breed showed 25 (14) markers in LR and 18 (4) markers in LW which were found to be chromosome wide (genome wide) significantly associated with one of the boar taint traits.

In LR an important region which contained 12 significantly associated markers with log_AND was identified on SSC 5 ranging from 20.9 Mb to 22.9 Mb. One of these associated markers (*ASGA0103650*) was a downstream gene variant of the gene *tachykinin 3* (TAC3). Although this gene seems to have a regulatory function in reproduction, it was excluded as a candidate gene by van Son et al. [45] because amino acid changes did not seem to have an effect on the protein function of TAC3. Nevertheless, significant associations with markers in this QTL and log_AND in fat were already described earlier in the study of Grindflek et al. [46] in Duroc. Close to this region Rowe et al. [47] reported a QTL for Danish Landrace boars for AND. Additionally, a QTL in this region was identified for testicular length and gonadosomatic index (GSI) by Große-Brinkhaus et al. [48]. It is described as an interesting, gene enriched region with possible candidate genes for AND biosynthesis [45]. In LW, one marker was found to be chromosome wide significantly associated with log_AND at 48.1 Mb on SSC 17. This variant is a 3′ prime untranslated region variant in a transcript region of the protein coding gene *PDX1 C-terminal inhibiting factor 1* (PCIF1). Until now, there are no information provided about this gene regarding consequences of mutations in pigs. Next to this region, significantly associated markers were found for AND [47] and SKA [48]. However, a few studies identified significant associations on SSC 17 in other regions for traits like average daily gain (ADG) in Italian LW pigs [49] or backfat thickness in LW and French LR populations [50]. In this study the LR breed showed more significant associations with log_AND than LW. Boars of both breeds were tested in the same age-dependent performance testing scheme (160 days) of the breeding company. However, due to the higher average daily gain (ADG) of 118.5 g/day of the LR pigs, sexual maturity within this breed was more expressed. This hypothesis is in accordance with the findings of Babol et al. [39] who proved the close relationship between ADG and begin of puberty. Beside this explanation, the higher amount of QTLs found for log_AND can be the result of breed differences, which were also postulated by Babol et al. [39].

In combination with the moderate to high h^2^ GWAS results confirmed the potential of breeding against AND, especially in LR. The region on SSC 5 seems to be important as has been shown by several authors [40, 42, 45–48]. Within this region no pleiotropic effects on maternal and paternal fertility can be found. Although GWAS did not show any regions for log_AND or log_SKA with pleiotropic effects on maternal and paternal fertility, results of variance component estimation indicate, that there is a common genetic background of the trait complexes boar taint and fertility.

For log_SKA there are significantly associated markers in both breeds that are located close to each other in a region on SSC 14 between 140.5 Mb and 141.6 Mb. One marker (*SIRI0000194*) was shared by both breeds as a genome wide significant upstream gene variant at position 141′690’183. This marker was also identified as the most significant SNP effect on SSC 14 for SKA in a study from Rowe et al. [47], although they used a prior version of the reference genome (*Sus scrofa* 10.2). The identified shared region lies within the promoter region of the CYP2E1 gene, which is described to be involved in the SKA metabolism in several crossbred and purebred lines [1, 51–54]. Although there is no indicator that CYP2E1 is involved in pathways linked to reproduction traits, CYP2E1 seems to be a promising across-breed candidate gene for enhancing the SKA metabolism.

Furthermore, nine chromosome wide significant markers for log_SKA were identified only for LR on SSC 6 between 0.3 Mb to 0.4 Mb and 5.5 Mb to 7.5 Mb. Within the last-named larger region, Ramos et al. [55] identified markers that were significantly associated with SKA. Furthermore, Grindflek et al. [46] characterized a breed specific QTL for SKA and Indole in Norwegian LR at the interval of 3.7–5.0 Mb on SSC 6. Additionally, several studies identified significant markers on this chromosome for AND [14, 46, 48, 56]. Grindflek et al. [14] identified a QTL for AND in Duroc on the same chromosome but in another region.

Other previously identified QTL regions for SKA or AND on SSC 6 in earlier studies [14, 48, 53, 56, 57] could not be confirmed by this study.

For paternal reproduction traits, no significant markers were identified. Taking into account the high h^2^ of these traits this result is somewhat unexpected and can be explained by a pure polygenetic inheritance of paternal fertility traits. But as has been mentioned above, boars with extremely negative fertility are not included within the data set. Along with the limited size of the genotype data set this could serve as a further explanation of the result of our study.

For maternal reproduction traits, GWAS identified significant markers for NBD and AFI in LR. The identified marker for NBD is an intron variant around 92.1 Mb on SSC 1 in a transcript of the protein coding gene *CD109 molecule* (CD109). As there is no link to fertility or boar taint for this gene, it can be excluded as a candidate gene. The marker which was significantly associated with AFI on SSC 1 is located at 0.4 Mb. This locus does not contain any gene. Another marker on SSC 2 was significantly associated with AFI in LR and is located at 11.7 Mb within the region of the gene *syntaxin 3* (STX3), which can be excluded as a candidate gene for AFI due to his functions and pathways.

In general, GWAS showed significant regions, which differed per breed, except for the shared region for log_SKA on SSC 14. Variance component estimation as well as GWAS indicated breed differences between LR and LW population. Variance component estimation showed that unfavorable relationships between boar taint and fertility could be possible. Multivariate approaches could be an appropriate tool to further investigate possible pleiotropic effects between boar taint compounds and maternal as well as paternal fertility.

## Conclusion

In conclusion, the results of the study showed contrary results for antagonistic relationships between boar taint and fertility in LR and LW breed. Therefore, the results could not serve as clear evidence that breeding for boar taint has relevant negative consequences for fertility traits in maternal breeds. In order to reduce boar taint, genomic selection in dam breeds for AND and SKA seems to be beneficial. Because no clear pleiotropic effects between boar taint and fertility were detected, this strategy is advisable without constraining effects of possible pleiotropic QTLs. However, detected antagonistic r_g_ between both trait complexes underline the necessity of a close monitoring of genetic changes. In case of unexpected genetic progress, selection intensity against boar taint should be lowered.

## Supplementary information

**Additional file 1: **Chromosome wide significant marker in LR after Bonferroni correction (*p* < 0.05). * = also genome wide significant (*p* < 0.05), n.m. = not mapped, UGV = upstream gene variant, DGV = downstream gene variant, SRV = splice region variant, I = intron variant, SYN = synonymous variant, IG = intergenic variant, 5’PUTR = 5′ prime UTR variant, 3’PUTR = 3′ prime UTR variant; Variance per SNP = explained phenotypic variance per significant SNP, SE = standard error.

**Additional file 2: **Chromosome wide significant marker in LW after Bonferroni correction (*p* < 0.05). * = also genome wide significant (*p* < 0.05), n.m. = not mapped, UGV = upstream gene variant, DGV = downstream gene variant, SRV = splice region variant, I = intron variant, SYN = synonymous variant, IG = intergenic variant, 5’PUTR = 5′ prime UTR variant, 3’PUTR = 3′ prime UTR variant; Variance per SNP = explained phenotypic variance per significant SNP, SE = standard error.

## Data Availability

All data sets used and analyzed during the current study are available from the corresponding author on reasonable request and with permission of the BHZP GmbH pig breeding company (henne@hbzp.de).

## Abbreviations

3’PUTR: 3′ prime untranslated region variant
5’PUTR: 5′ prime untranslated region variant
ADG: Average daily gain
AFI: Age at first insemination
AI: Artificial insemination
AND: Androstenone
CD109: CD109 molecule
CO_2_: Carbon dioxide
CYP2E1: Cytochrome P450 2E1
DGV: Downstream gene variant
g: Gram
GC: Genomic control
GnRH: Gonadotropin-releasing hormone
GSI: Gonadosomatic index
GWAS: Genome wide association analysis
h^2^: Heritability
I: Intron variant
IG: Intergenic variant
kg: Kilogram
LH: Luteinizing Hormone
log_AND: Log-transformed Androstenone
log_SKA: Log-transformed Skatole
LR: Landrace
LW: Large White
MAF: Minor allele frequency
Mb: Mega base pairs
ml: Milliliter
n.m.: Not mapped
N: Number
NBA: Number of piglets born alive per litter
NBD: Number of piglets born dead per litter
ng: Nanogram
OD: Optical density
PCIF1: PDX1 C-terminal inhibiting factor 1
QTL: Quantitative trait locus
r_g_: Genetic correlation
r_p_: Phenotypic correlation
SC: Sperm count
SE: Standard Error
SIDA-HSPM-GC/MS: Standardized stable isotope dilution analysis-headspace solid-phase microextraction-gas chromatography/mass spectrometry
SKA: Skatole
SNP: Single nucleotide polymorphism
SP: Sperm density
SRV: Splice region variant
SSC: *Sus scrofa chromosome*
STX3: Syntaxin 3
SV: Sperm volume
SYN: Synonymous variant
TAC3: Tachykinin 3
UGV: Upstream gene variant
UTR: Untranslated region
